# Prevalence and Types of Congenital Anomalies in Singleton Pregnancies at a Tertiary Care Hospital

**DOI:** 10.7759/cureus.76506

**Published:** 2024-12-28

**Authors:** Uzma Manzoor, Muhammad Saad, Sadaqat Ali, Sadia Bano, Uzma Shahzad, Muhammad Zain

**Affiliations:** 1 Obstetrics and Gynecology, Independent Medical College, Faisalabad, PAK; 2 Internal Medicine, Ameer-ud-Din Medical College, Lahore, PAK; 3 Internal Medicine, Aziz Fatimah Hospital, Faisalabad, PAK; 4 Obstetrics and Gynecology, Shalamar Medical and Dental College, Lahore, PAK

**Keywords:** advanced maternal age, cleft lip and palate, congenital anomalies, congenital malformations, folic acid supplementation, gestational diabetes mellitus (gdm), neural tube defects (ntds), perinatal morbidity, polyhydramnios, pregnancy

## Abstract

Introduction

Congenital malformations are a major cause of perinatal morbidity and mortality in developing countries and are assuming greater importance than ever before. They affect a variety of organ systems and various etiologies have been identified in literature including Toxoplasmosis, Other (syphilis, varicella-zoster, parvovirus B19), Rubella, Cytomegalovirus, Herpes Simplex (TORCH) infections, exposure to pollutants, consumption of tobacco and alcohol, and advanced maternal age. In developing countries, diagnosis is frequently delayed which leads to poorer outcomes.

Method

All patients with a singleton pregnancy, with either a diagnosis of congenital anomaly from 28 to 37 weeks of gestation on ultrasonography (USG) or the discovery of an anomalous fetus after delivery were selected.

Results

Out of 572 patients delivered in one year, 29 (5.1%) delivered babies with congenital anomalies. Seventeen (59%) patients with a congenitally anomalous fetus were primigravida and 18 (62.1%) belonged to the age group >35 years. Twenty-three (79.3%) patients were diagnosed with congenital anomalies on USG at a gestational age of 28-37 weeks. The most common associations with congenital anomalies were advanced maternal age (37.9%), gestational diabetes mellitus (GDM) (20.6%), and lack of folic acid intake (10.3%). The most common types of congenital anomalies were central nervous system (CNS) anomalies (41.4%), musculoskeletal anomalies (20.6%), and gastrointestinal tract (GIT) anomalies (17.2%).

Conclusion

Neural tube defects are the most common and can be prevented by ensuring folate supplementation, especially during the first trimester. As part of family planning, new couples should be counseled regarding the risks of advanced maternal age. For diabetic mothers, emphasis should be placed on glycemic control and dietary restrictions.

## Introduction

Congenital malformations (CMs) or birth defects are defined as alterations in the structure, function, or metabolism of organ systems of a newborn leading to physical and mental disabilities or even death [1]. Globally the incidence of CMs varies depending upon the population under study. In Karachi, Pakistan, Korejo et al. reported 6.2% of perinatal deaths due to CMs [2]. Infectious disease which was once a major cause of perinatal mortality in developing countries now has a lesser contribution due to improved access to healthcare [3]. CMs now constitute a greater percentage of infant deaths than ever before, thus assuming greater medical importance. CMs can affect many organ systems depending upon the stage of embryogenesis, e.g., central nervous system (CNS), gastrointestinal tract (GIT), musculoskeletal (MSK) system, etc. The etiology of CMs is multifactorial, e.g., maternal Toxoplasmosis, Other (syphilis, varicella-zoster, parvovirus B19), Rubella, Cytomegalovirus, Herpes Simplex (TORCH) infections, exposure to pesticides, environmental pollutants, radiation, tobacco and alcohol use, advanced maternal age, and various medical conditions in the mother [4]. Diagnosis of CMs occurs relatively late in developing countries such as ours with many cases only being diagnosed after birth [5]. The advent of advanced imaging techniques allows for earlier diagnosis and prompt intervention. This study was conducted because there have been few studies from Pakistan, and even fewer from Faisalabad, documenting the incidence of CMs. We aimed to identify the frequency of CMs and their etiological factors in neonates born in a tertiary care hospital in Faisalabad. The findings of our study could then be used to guide public health interventions by authorities in resource-limited countries like Pakistan.

## Materials and methods

This was a descriptive, cross-sectional, observational study. The study was conducted at Independent University Hospital, Faisalabad, a tertiary care hospital associated with Independent Medical College, from January 2021 to December 2022. The institutional review board (IRB) approval was obtained from the ethical committee of the Independent University Hospital under reference number IUH/IRB/000044. Out of 572 total walk-in patients, 29 (5%) were selected. Inclusion criteria were as follows: singleton pregnancy, CM diagnosed on USG from 28 to 37 weeks of gestation, and delivery of an anomalous fetus. To diagnose CMs on ultrasound, we looked for anatomical defects such as shortened/malformed bones, tissues, and organs. The presence of gross anatomical defects on physical inspection was used to diagnose CMs after delivery. Patients diagnosed with CMs before 28 weeks of gestation and those with twin pregnancies were excluded from the study. Informed consent was taken from all patients involved in the study. All patient data were kept confidential with access exclusively reserved for researchers involved in this study. WHO sample size calculator was used, taking a confidence interval of 95% with an incidence of congenital anomalies of 2-4% and a margin of error of 7% [6]. Detailed questioning regarding gynecological and obstetric history, drug history, history of medical disorders, cousin marriage, nutritional status including folic acid intake, general physical examination, detailed USG examination, and routine investigations were carried out. Congenital anomalies were identified either on USG or after delivery. Data collection was performed via structured forms specifically designed for this study. Data were analyzed using SPSS version 23 (Armonk, NY: IBM Corp.). Quantitative variables like age, gestational age, parity, frequency, and percentage were calculated. Qualitative variables were gender, live births, and congenital malformations.

## Results

Out of a total of 572 walk-in patients who underwent delivery, 543 delivered normal fetuses and 29 (5.1%) delivered anomalous fetuses. Out of these 29 patients, 17 (59%) were primigravida and 12 (41%) were multigravida. Patients were distributed into two age groups as follows: <35 years age group (11, 37.9% patients) and >35 years age group (also defined as advanced maternal age) (18, 62.1% patients), thus showing a positive correlation of maternal age with the frequency of CMs (Table 1). USG examination is a routine part of prenatal care. Most women receive at least two USG scans throughout pregnancy, one in the first trimester and one in the second. Obstetricians may also choose to conduct further USG scans in case of clinical suspicion of pathology or maternal/fetal complications. In the present study, 23 (79.3%) patients were diagnosed with CMs at gestational age 28-37 weeks using USG.

**Table 1:** Distribution of patients according to presence of fetal anomaly, gravidity, and age group.

Variables		No. of patients	Frequency
Presence of fetal anomaly	Normal fetus	543	94.9%
	Anomalous fetus	29	5.1%
Gravidity	Primigravida	17	59%
	Multigravida	12	41%
Age group	<35 years	11	37.9%
	>35 years	18	62.1%

A detailed history was obtained from all patients. This in combination with the clinical record of the patients was used to identify potential causes of CMs (Table 2). The most common etiology was advanced maternal age in 11 (37.9%) patients, followed by gestational diabetes with polyhydramnios in six (20.6%) patients, lack of folic acid intake during pregnancy in three (10.3%) patients, and cousin marriage in two (6.9%) patients. No discernible cause was found in seven (24.1%) patients.

**Table 2:** Distribution of patients according to potential etiological factors.

Etiological factor	No. of patients	Frequency
Advanced maternal age	11	37.9%
Idiopathic	7	24.1%
Gestational diabetes with polyhydramnios	6	20.6%
Lack of folic acid intake	3	10.3%
Cousin marriage	2	6.9%

**Figure 1:**
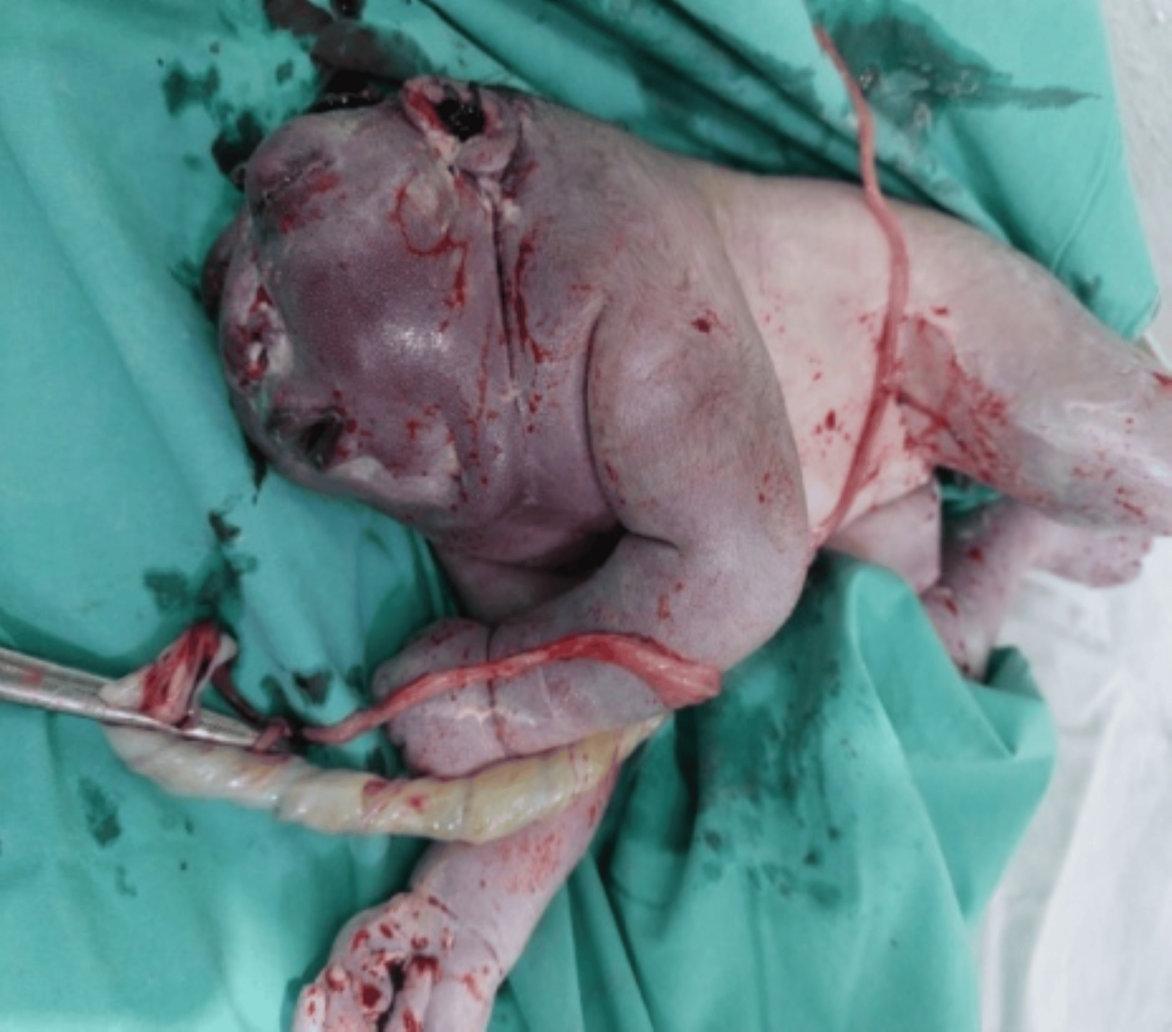
Absence of a major portion of the brain and cranial vault. Anencephaly is a neural tube defect characterized by defective development or absence of a major portion of the cranial vault, scalp, and/or cerebral hemispheres.

**Figure 2:**
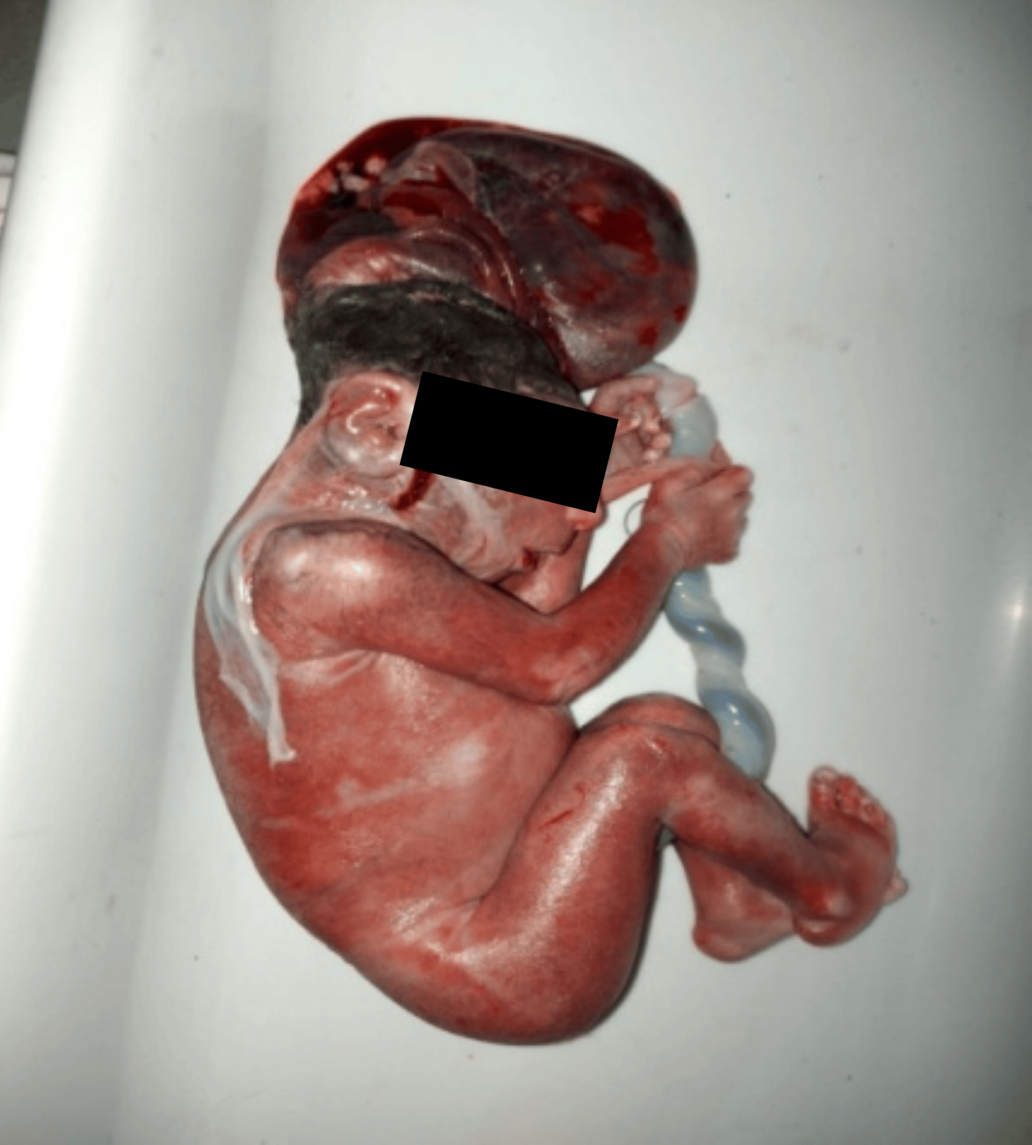
Herniation of brain tissue through a defect in the cranial vault. Encephalocele is a congenital neural tube defect characterized by the herniation of intracranial contents, including brain tissue and cerebrospinal fluid, through a defect in the cranial vault.

Multiple organ systems were found to be affected in the congenitally anomalous fetuses. Neural tube/CNS defects were most commonly found in 12 (41.4%) fetuses (Table 3). Figure 1 shows one of these fetuses with anencephaly. Anencephaly is the second most common neural tube defect (NTD) globally and affected fetuses lack development of a major portion of the cranium, scalp, and brain. The condition is almost invariably fatal as was in the present case. Another case of NTDs was of encephalocele, a malformation characterized by protrusion of brain tissue covered with membranes (Figure 2).

**Table 3:** Distribution of patients according to type of fetal anomaly. GIT: gastrointestinal tract

Type of abnormalities	No. of fetuses	Frequency
Neural tube/CNS defects	12	41.4%
Musculoskeletal defects	6	20.6%
GIT defects	5	17.2%
Urogenital defects	3	10.3%
Congenital Heart defects	2	6.9%
Other genetic disorders	1	3.4%

**Figure 3:**
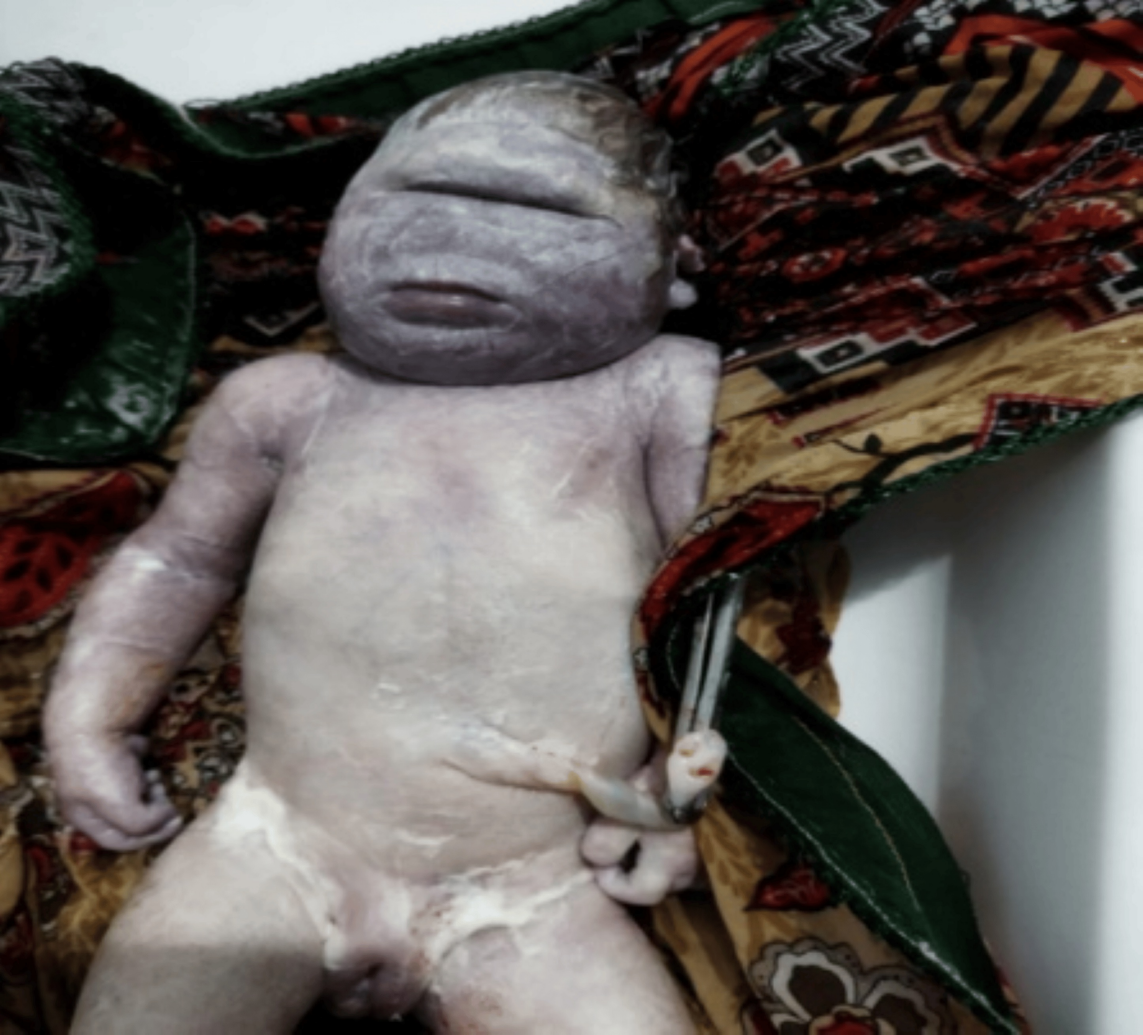
Failure of cleavage of the forebrain with associated cyclopia. Holoprosencephaly is a congenital malformation resulting from incomplete forebrain cleavage during early embryonic development, leading to varying degrees of brain and facial abnormalities. It often presents with facial dysmorphology and can range from mild to severe, depending on the extent of the brain division failure.

**Figure 4:**
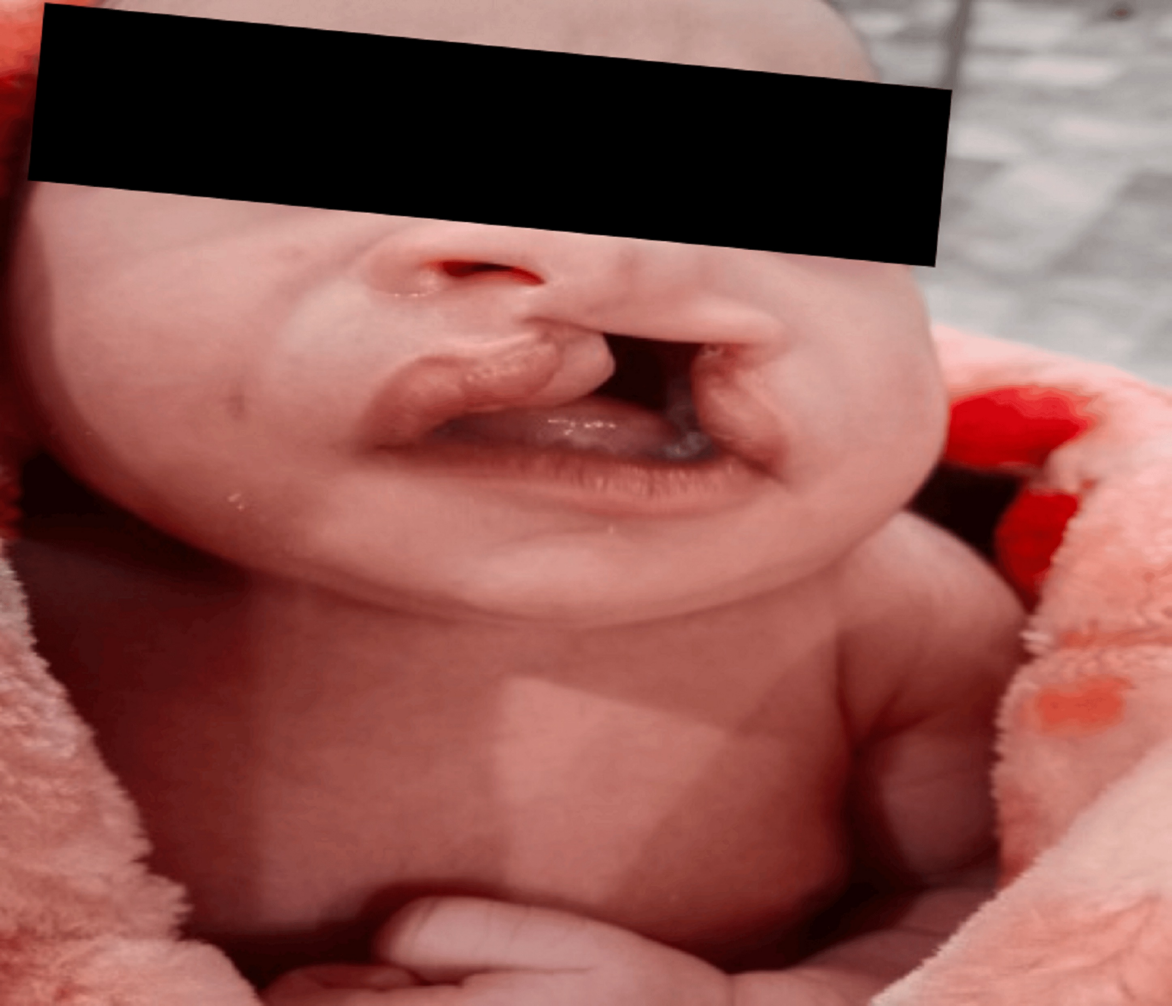
Failure of fusion of maxillary and palatal structures leading to a defect in the lip and palate. Cleft lip and palate are congenital malformations caused by incomplete fusion of the maxillary and palatal structures during embryogenesis. They can lead to functional impairments such as feeding, speech, and hearing issues.

**Figure 5:**
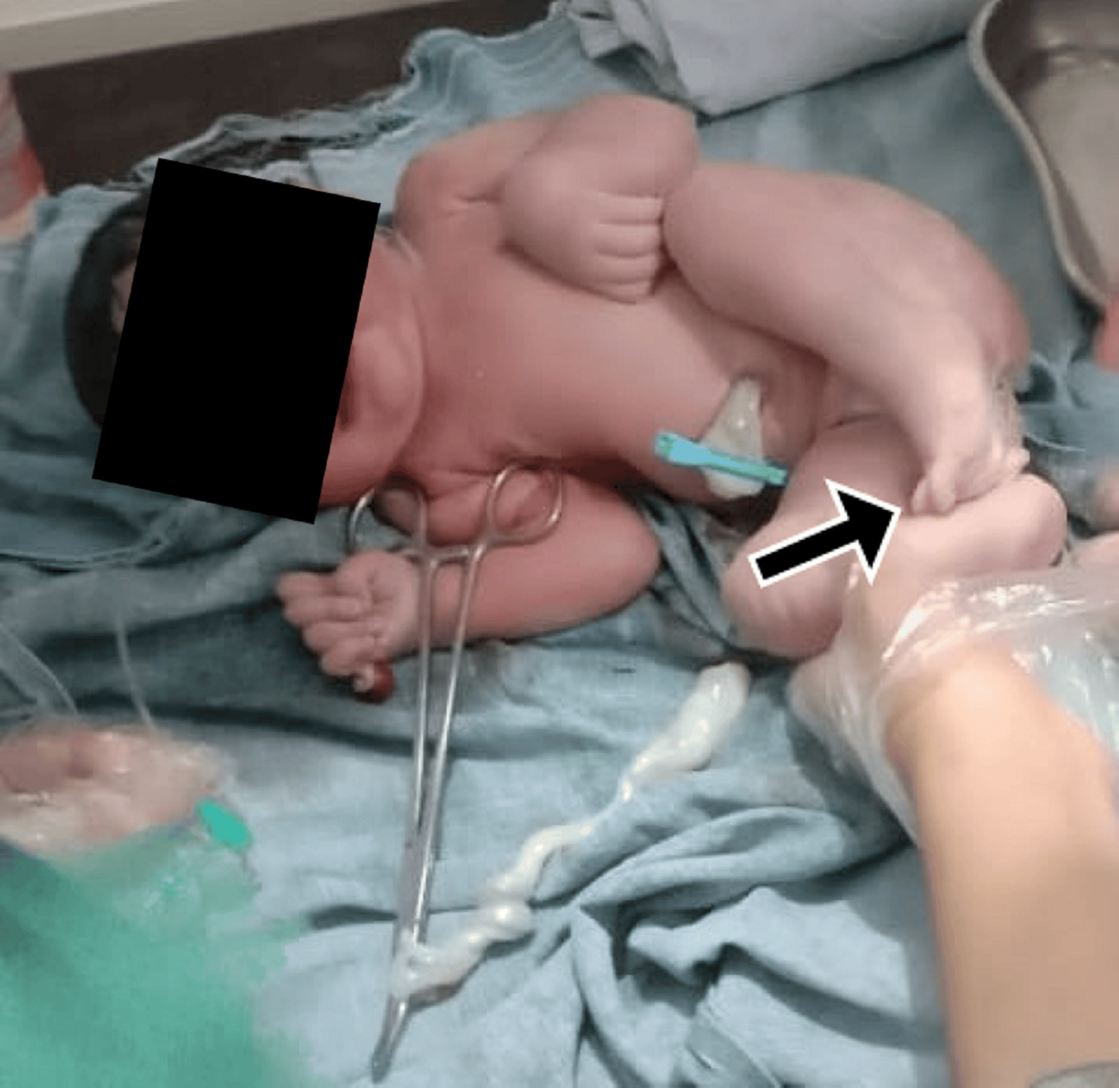
Deformed left foot and toes along with complete absence of right foot. Skeletal dysplasia encompasses a heterogeneous group of genetic disorders resulting from mutations affecting the development and growth of bone and cartilage, leading to structural skeletal abnormalities. These conditions, which include osteochondrodysplasias, such as achondroplasia and osteogenesis imperfecta, vary in severity, with manifestations ranging from dwarfism to lethal perinatal forms.

Prognosis varies depending upon the size of the defect and amenability to surgical correction. A third case was holoprosencephaly, which entails a failure of the embryonic prosencephalon to develop into two hemispheres along with defective development of the face, eyes, nose, or upper lip (Figure 3). The second most common type of CMs was musculoskeletal with six (20.6%) fetuses (Table 3). Figure 4 shows a case of cleft lip and palate, one of the most well-recognized and common MSK defects. Similarly, Figure 5 shows a case of lower limb skeletal dysplasia. A black arrow indicates the total absence of a right foot along with a deformed left foot. MSK anomalies were followed by GIT anomalies in five (17.2%) fetuses, urogenital anomalies in three (10.3%) fetuses, and congenital heart defects in two (6.9%) fetuses (Table 3).

## Discussion

In the present study, conducted over one year, 29 out of a total of 572 patients delivered babies with CMs. Thus, the frequency of congenitally anomalous fetuses was 5.1%. This was lower than the 6.2% prevalence reported by Korejo et al. in Karachi, Pakistan [2], but higher than the figure of 4.2% reported by Gillani et al. in Abbottabad, Pakistan [7]. These differences among studies conducted in the same country could be explained by their sample populations belonging to different ethnicities, i.e., Punjabi, Sindhi, and Pathan ethnicities. Prior research has shown that race and ethnicity do influence the occurrence of genetic disorders within a locality [8]. A glance at studies from other developing nations shows that Ajao and Adeoye in Nigeria reported a prevalence of congenitally anomalous fetuses of 6.3% [9] and Mekonen et al. in Ethiopia reported a frequency of 3.13% [10]. Hameed in Baghdad, Iraq, reported a frequency of 1.2% [11], and an extensive 20-year-long study conducted in our neighboring India reported a frequency of 1.82% [12]. This shows that the prevalence of CMs varies depending on the population under study.

Most of our patients were primigravida. This presents an additional challenge for first-time mothers as they are often unprepared for the challenges of childbirth [13]. The birth of an anomalous fetus adds to their woes and makes for a particularly traumatic experience. Efforts should be made to ascertain if there is a genetically inheritable cause for the anomaly. This enables couples to make an informed decision regarding the risk of fetal CM in future pregnancies. Mothers may also have feelings of guilt or shame at the birth of an anomalous fetus [14]. Obstetricians should alleviate these feelings and explain in no uncertain terms that fetal CMs are not the result of anything mothers did during pregnancy or beforehand.

Twenty-three (79.3%) patients in the present study were diagnosed with USG during pregnancy as our center is adequately equipped with modern imaging modalities. This may not be the case for other hospitals in developing countries due to the relatively high cost of imaging equipment, especially in rural areas. Early diagnosis of CMs is especially important given the significant associated perinatal morbidity and mortality and the immense expense associated with the treatment and rehabilitation of such babies [15]. Earlier diagnosis allows for better parental counseling and psychological readiness for associated challenges. It also enables physicians to offer timely interventions such as termination of pregnancy or specialist referral [3].

The majority of patients in our study were of age >35 years. These results were comparable to Sadiq et al. [16] and Anele et al. [17]. Advanced maternal age (AMA), defined as age >35 years, has been consistently associated with gestational diabetes and hypertension along with poor perinatal outcomes, e.g., low APGAR score, preterm birth, and low birth weight [18,19]. The association of AMA with CMs is partly due to the increased occurrence of aneuploidies and chromosomal defects [20]. However, non-chromosomal abnormalities, especially cardiac CMs, have also been found to increase with AMA indicating a non-chromosomal pathophysiology [21].

Six (20.6%) patients in the present study had gestational diabetes with polyhydramnios. Gestational diabetes mellitus (GDM) and pre-pregnancy DM, both are associated with an increased frequency of CMs [22]. While glycemic control was not directly measured in our study, existing literature does show a link, and the pathophysiology is postulated to involve a combination of factors brought about by maternal hyperglycemia such as oxidative stress, apoptosis of fetal cells, teratogenesis, and epigenetic changes [23]. Diabetic mothers should be counseled regarding the adverse effects of hyperglycemia on fetal health. Physicians should also emphasize tight glycemic control and dietary precautions. For seven (24.1%) patients, no discernible cause was found. An overview of existing literature offers insight into possible causes for these cases. Environmental pollution, especially air pollution, is widespread in developing countries and has been implicated in the development of CMs along with a host of other respiratory diseases [24]. Public health policy should focus on controlling sources of environmental pollution. Pregnant mothers should be advised to avoid polluted areas and wear masks in such conditions. Other common causes of CMs are the infamous maternal TORCH infections which cause a multitude of problems including sensorineural deafness, patent ductus arteriosus (PDA), cataracts, and CNS anomalies [25]. These CMs can be prevented by employing measures such as maternal vaccination, safe sex practices, and timely diagnosis and treatment. Our study showed AMA and DM as the first and third most common factors for CMs. Public health policy should include couples counseling that recommends childbirth at maternal ages below 35 years. Primary care interventions to reduce DM such as as dietary modifications and exercise may also prove beneficial.

Neural tube defects were the most common type of CM which is congruent with the findings of Ameen et al. in Iraq [26]. Deficiency of folic acid, especially in the first trimester which is critical for organogenesis, has frequently been implicated in the development of NTDs. Inadequate folate intake was found in three (10.3%) patients. NTDs can thus be prevented through antenatal folic acid supplementation [27]. Maternal counseling is imperative in this regard and obstetricians should inquire frequently about adherence to folate supplements in scheduled follow-up visits. MSK defects were the second most common on the list. This is a cause of concern as these are frequently non-fatal and the surviving child is burdened with lifelong physical and movement disability. Although advancements in limb prosthetics and surgical techniques for these conditions have improved the prognosis for such patients, these modalities are not curative, and significant residual impairment persists. In another study by Ekwunife et al., GIT CMs were the most common [28]. These include omphalocele, gastroschisis, anorectal anomalies, and tracheoesophageal fistulas. Cherian et al. reported MSK CMs as the most common in their population [29].

As this cross-sectional study was performed in a single tertiary care center with a relatively small sample size, its results may not accurately reflect the population as a whole. Multicenter studies with a larger sample size and prospective cohort studies are necessary in this regard. Due to resource constraints, our study relied on history taking to assess etiological factors, such as environmental pollution, diet, family history, and genetic predisposition. This method is susceptible to recall bias and further studies performed under more controlled conditions, such as dietary and environmental monitoring and genetic testing, are needed. Cross-verification of information through other methods can also reduce the effect of recall bias.

## Conclusions

Emphasis should be placed on raising awareness about folic acid intake in the first trimester to prevent NTDs, maintaining good glycemic control in GDM, formulating public health policies aimed at avoiding exposure to environmental toxins, and ensuring timely vaccination. By employing such measures, the prevalence of congenital fetal anomalies and the associated perinatal morbidity and mortality can be significantly reduced. Obstetricians should also aim for early diagnosis of CMs during pregnancy to facilitate referral for specialist management.
